# MicroRNA Profiling of the Effect of the Heptapeptide Angiotensin-(1-7) in A549 Lung Tumor Cells Reveals a Role for miRNA149-3p in Cellular Migration Processes

**DOI:** 10.1371/journal.pone.0162094

**Published:** 2016-09-06

**Authors:** Brenda de Oliveira da Silva, Kelvin Furtado Lima, Letícia Rocha Gonçalves, Marina Bonfogo da Silveira, Karen C. M. Moraes

**Affiliations:** 1 Núcleo de Pesquisa em Biologia, Universidade Federal de Ouro Preto, Ouro Preto, MG, Brazil; 2 Institute of Chemistry, Universidade Estadual Paulista “Júlio de Mesquita Filho”, Araraquara, SP, Brazil; 3 Molecular Biology Laboratory, Departament of Biology, Bioscience Institute, Universidade Estadual Paulista “Júlio de Mesquita Filho”, Rio Claro, SP, Brazil; Michigan State University, UNITED STATES

## Abstract

Lung cancer is one of the most frequent types of cancer in humans and a leading cause of death worldwide. The high mortality rates are correlated with late diagnosis, which leads to high rates of metastasis found in patients. Thus, despite all the improvement in therapeutic approaches, the development of new drugs that control cancer cell migration and metastasis are required. The heptapeptide angiotensin-(1–7) [ang-(1–7)] has demonstrated the ability to control the growth rates of human lung cancer cells in vitro and in vivo, and the elucidation of central elements that control the fine-tuning of cancer cells migration in the presence of the ang-(1–7), will support the development of new therapeutic approaches. Ang-(1–7) is a peptide hormone of the renin-angiotensin system (RAS) and this study investigates the modulatory effect of the heptapeptide on the expression pattern of microRNAs (miRNAs) in lung tumor cells, to elucidate mechanistic concerns about the effect of the peptide in the control of tumor migratory processes. Our primary aim was to compare the miRNA profiling between treated and untreated-heptapeptide cells to characterize the relevant molecule that modulates cellular migration rates. The analyses selected twenty one miRNAs, which are differentially expressed between the groups; however, statistical analyses indicated miRNA-149-3p as a relevant molecule. Once functional analyses were performed, we demonstrated that miRNA-149-3p plays a role in the cellular migration processes. This information could be useful for future investigations on drug development.

## Introduction

Lung cancer is one of the most frequent types of cancer in humans and a leading cause of death in both men and women worldwide, accounting for over 1.59 million deaths in 2012 [1]. Tobacco use still accounts for 80–90% of the lung cancer cases; however, occupational exposures to carcinogens account for approximately 9 to 15 percent of the cases and outdoor air pollution is responsible for 1 to 2 percent of affected individuals [2,3]. There are two main types of lung cancer: the non-small cell lung cancer (NSCLC) and the small lung cancer (SLC). The NSCSL is responsible for approximately 85% of the cases, with subtypes squamous cells carcinoma, adenocarcinoma, and large cell carcinoma. Although, the SLC affects only ~15% of patients, this type of cancer can spread quickly. Adenocarcinoma represents about 40% of lung cancers and they normally start in mucus-secreting cells. This type of lung cancer is more frequently found in women, more likely to occur in young people and usually occurs in the outer parts of the lung [4,5].

Over the past few years, an increased number of NSCLC patients who had never smoked have been observed [6]. This demands the attention of health organizations worldwide and the need to develop alternative therapies for treatment of patients. Despite all the improvement in the therapeutic approaches, the 5-year survival rate of patients with lung cancer is around 10%, with many new cases of the disease diagnosed annually. The high mortality rates are correlated with the late diagnosis, which lead to high rates of metastasis found in patients [7]. Thus, the control of cellular migration and metastasis could help to improve the lung cancer treatment and patients’ life expectancy.

To support the development of new therapies for lung cancer, several studies have been performed. In more recent years, the heptapeptide angiotensin-(1–7) [ang-(1–7)] has demonstrated the ability to control the growth rates of human lung cancer cells in vitro, reduce the size of human lung tumor xenografts in vivo [7,8,9] and decrease tumor vascularization [3]. This peptide mediates biological functions through activation of its G-protein coupled receptor, Mas [10], which acts on multiple layers of molecular mechanisms that control cellular equilibrium. Ang-(1–7) is a peptide hormone of the renin-angiotensin system (RAS) and was described as an important element correlated with the control of the cardiovascular system [11,12]. Its modulatory activity on cancer growth has been indicated as a promising therapy [13]; however, further studies are needed on the mechanistic details of such modulatory effect of the heptapeptide on tumor behavior. Particularly, many molecular interplays in a tumor cell support migration and metastasis. However, the effects of ang-(1–7) in the control of such cellular mechanisms have not been fully investigated. Therefore, unravelling the central elements that control the fine-tuning of cancer cells in the presence of the ang-(1–7) will contribute to the development of new therapeutic approaches.

In the present study, we decided to investigate the modulatory effect of the heptapeptide on the expression pattern of microRNAs (miRNAs) in lung tumor cells, to elucidate physiological concerns about the effect of the peptide in controlling tumor migration. The relevance of this cellular physiological process is supported by the fact that metastatic tumor diseases are largely incurable, considering their systemic behavior, which promotes high mortality rates [14]. Hence, our primary aim was to compare the miRNA profiling between treated and untreated-heptapeptide in NSCLC cells to characterize the relevant molecule that modulates cellular migration rates. The analyses selected twenty one miRNAs, which are differentially expressed between the groups. Statistical analyses indicated miRNA-149-3p as a relevant molecule and functional analyses demonstrated that this miRNA plays a role in the cellular migration processes. Thus, the innovative study is a promising investigation for future therapeutic strategies.

miRNAs are small (21–25 nt) non-coding RNAs that regulate gene expression by binding to the 3’-untranslated region (UTR) of target mRNAs, which decreases the protein synthesis [15,16,17]. These molecules have emerged as a powerful element that may affect various phenotypes such as disease susceptibility and drug response [18]. Several reports have demonstrated that changes on miRNA level are strictly correlated with the physiological or pathological states of the cells [19], which provide new biomarkers for the outcome of diseases. In addition, large efforts have been made by pharmaceutical companies to develop the RNA-based therapeutics for several diseases including cancer. However, a systemic view of the effect of these molecules and their major biochemical interconnections should be completely understood before pre-clinical trials. Thus, considering the modulatory effect of the ang-(1–7) in tumor behavior, uncovering the molecular interplays of its effect on migration process is relevant.

## Materials and Methods

### Cell culture procedure

The NSCLC A549cells (ATCC® CCL-185™) were maintained in Dulbecco´s Modified Eagle Medium/ Nutrient Mixture F-12 (DMEM/F12) supplemented with 4 mM of L-glutamine and 10% of fetal serum bovine (FBS) at 37°C in an atmosphere that contained 5% CO_2_. NCI-H460 (ATCC® HTB-177™, a NSCLC) and MRC5 (ATCC® CCL171™, a normal cell line) cells were grown in Roswell Park Memorial Institute (RPMI) 1640 medium or Dulbecco’s Modified Eagle Medium (DMEM), respectively, and those cells were maintained at the same temperature and atmosphere in the presence of 10% of FBS. For experiments, cells were seeded in specific plates at the density of 3.2 x 10^3^ cells/ cm^2^, and the heptapeptide ang-(1–7) (Bachem Americas Inc, USA) was added or not to the cultures at a final concentration of 10^−7^ M [20,21]. The media was renewed every 24 h and the cells were used in the assays when the cultures reached ~90% confluency.

### Total RNA isolation and miRNA profiling

Total RNA from ang-(1–7)-treated or untreated cell culture was isolated using mirVana™ mRNA isolation kit (Ambion™, Thermo Fisher Scientific, USA) and the quality of the samples were analyzed on 8% denaturing urea polyacrylamide gel. Next, Bioanalyzer (Agilent Technologies, California, USA) was used to calculate the RIN value for RNA. Samples with RIN value more than 9.2 were used in the subsequent set of analyses. Next, miRNA reversed transcription and the array profiling were performed following the instructions of the miRCURY LNA™ Universal RT microRNA PCR array (Exiquon, Denmark), which targets 776 human miRNAs registered in the miRBase version 20.0 at the Sanger Institute. Real time PCR (qPCR) reactions were processed in a ViiA™ 7 Real-Time PCR System (Thermo Fisher Scientific, USA). The experiments were performed in triplicate.

### Data analysis

The analyses of the microRNA arrays identified the upregulated and the downregulated miRNAs differentially expressed in A549 cell cultures. The qPCR analyses were performed using the Exiqon GenEx software version 3.0 following the provider’s directions. For the expression analyses, the results from each separated PCR panel were calibrated between themselves, by performing a common amplification in all plates (Inter-Plate Calibrator, IPC), which compensates for the bias introduced in different runs. As recommended by Exiqon and GenEx, the cut off value of a threshold cycle (Ct) was set at 39. Next, data were normalized using the global mean of all expressed miRNA, and the values obtained correspond to the ΔCt. Differences in miRNAs expression between treated and untreated cells with the heptapeptide were compared by t-statistics with Bonferroni correction (GenEx software). One-Way ANOVA and post hoc Fisher LSD was also applied to the data analyses in order to allow detection of possible false negative from Bonferroni correction.

In addition, Pearson correlation test was used to analyze the correlations between the miRNAs, which present significantly different expression in the investigated cellular groups. For the heat map construction, only clustering of miRNAs with significantly different expression (p ≤ 0.001) between the samples were used. The correlation plot and the heat map were generated using GenEx software.

### Real time PCR validation

The miRCURY LNA™ PCR array results were validated by real time PCR using ExiLENT SYBR® Green master mix (Exiquon, Denmark) and specific set of miRCURY LNA™ primers for has-miR-149-3p, has-miR-486-5p, has-miR-483-3p, has-miR-34b-5p, has-miR-99b-3p, has-miR-140-5p, has-636 and has-miR-495-3p, all of them from Exiquon manufacture. qPCR reactions were performed in a StepOne™ Real-Time PCR System (Thermo Fisher Scientific, USA) and to normalize the expression values, a small nuclear RNA with constitutive expression, U6, was used as reference gene. The expression level was quantified using the 2 ^-ΔΔCT^. All values were plotted relative to the normal control values, which were represented as a value of 1.

### Selection of miRNAs and genes

To select the miRNA that was functionally investigated in this study, statistical correlation was considered between the groups, and the highest differentially expressed miRNA after the treatment with the heptapeptide was chosen for future analyses. In addition, using miRBase and TargetScan public databases, we identified 26 potential targets for the selected miRNA, based on their role in the migration processes described in the literature searched at PubMed and Web of Science. The information available at the Kyoto Encyclopedia of Genes and Genomes (KEGG) was also analyzed. The expression levels of the selected genes were evaluated by qPCR using specific set of primers and relative quantification using the 2 ^-ΔΔCT^ for all the investigated conditions. Table 1 presents the selected genes and the potential target score for the investigated miRNA.

**Table 1:** List of potential target genes and their respective target score for the highest differentially expressed miRNA after cellular treatment with the angiotensin-(1–7).

GENE SYMBOL	GENE DESCRIPTION	TARGET SCORE
AAMP	angio-associated, migratory cell protein	73%
ADAM11	ADAM metallopeptidase domain 11	53%
CADM1	cell adhesion molecule 1	65%
CADM3	cell adhesion molecule 3	78%
CLDN10	Claudin 10	53%
COL1A1	collagen, type I, alpha	82%
COL1A2	collagen, type XI, alpha 2	79%
ELN	elastin	90%
ITGA1	itegrin, alpha 1	56%
ITGA2B	integrin, alpha 2b	77%
ITGA3	integrin, alpha 3	69%
ITGA5	integrin, alpha 5	67%
LAMC1	laminin, gamma 1	94%
LAMC3	laminin, gamma 3	72%
MMP2	gelatinase—matrix metallopeptidase 2	78%
MMP14	matrix metallopeptidase 14	82%
MMP15	matrix metallopeptidase 15	59%
PDGHGA10	protocadherin gamma subfamily A, 10	64%
PCDHGC3	protocadherin gamma subfamily C, 3	76%
PCDHGC5	protocadherin gamma subfamily C, 5	65%
PCDHGB7	protocadherin gamma subfamily B,7	64%
SMAD2	SMAD family member 2	65%
SMAD3	SMAD family member 2	82%
TIMP2	TIMP metallopeptidase inhibitor 2	85%
TIMP3	TIMP metallopeptidase inhibitor 3	97%
TJAP1	tight junction associated protein 1	67%

The selected genes are correlated with the biological process of cell migration.

### Cellular transfections with miRNA mimics and inhibitors

To identify optimal transfection conditions for the assays, transfection efficacy was also evaluated by fluorescence microscopy, using different concentrations of Blockit Alexa Fluor Red Oligo (Thermo Fisher Scientific, USA) and different volumes of the transfection reagent Lipofectamine® 2000 Transfection Reagent (Thermo Fisher Scientific, USA). After the initial trial, positive and negative mirVana™ miRNA mimic (miR-1 Positive Control) and inhibitor (Negative Control #1) from Thermo Fisher Scientific were used at different concentrations, along with1 μl of the transfection reagent following the supplier’s instructions. After 48 h transfection, the efficacies of the assays were evaluated by qPCR for the specific target gene of the controls, *PTK9*, and cellular viability was measured using Trypan blue staining–(S1 Fig). The combined results were useful to establish optimal concentration of mirVana™ molecules for functional assays. The same procedures were performed to optimize the best cellular transfection conditions for the two other cell lines (NCI-H460 and MRC5) used in this study (data not shown).

For each transfection assay, 6x10^4^ cells were cultured overnight under regular growth conditions in a 24-well plate. Next, transfections were performed using 10 nM of miRNA specific mimic or inhibitor according to the supplier instruction of the Lipofectamine® 2000 Transfection Reagent (Thermo Fisher Scientific). After 48 h, cellular and molecular events that underlie migration processes in transfected cells were evaluated.

### Immunofluorescence and phase-contrast microscopy

A549 investigated cells were fixed in 3.8% paraformaldehyde solution containing 0.2% Triton X-100 (Sigma-Aldrich, USA) for 5 min at 37°C and subjected to immunostaining. To evaluate the effect of angiotensin-(1–7) and miRNA transfections in A549 cellular morphology, the actin filaments were stained in a 1% bovine serum albumin (BSA) solution containing 100 μg/ ml of phalloidin-tetramethylrhodamine B isothiocyanate (TRITC) (Sigma-Aldrich, USA) for 1 h. Next, the nuclei of the cells were counterstained in 3.33 ng/ ml 4’,6 diamino-2-phenylindole (DAPI, Sigma-Aldrich, USA) solution for 5 min. After extensive washes with saline phosphate buffer (PBS, 2.7 mM KCl, 1.5 mM KH_2_PO_4_, 137 mM NaCl, and 8 mM Na_2_HPO_4_, pH 7.4), the coverslips containing cells were subsequently mounted onto slides and subjected to microscopic immunofluorescence analysis. Images were obtained with an Olympus BX51 microscopy equipped with corresponding filter sets and a DP71 CCD camera (Tokyo, Japan). One hundred randomly selected cells were analyzed in each investigated group. For phase-contrast analyses required in the in vitro scratching assays (wound healing) and in the cellular invasion chamber assays, the same microscopy was used and images were monitored and quantified using the Image J software (http://rsb.info.nih.gov/ij/).

### Rna interference assay

The predesigned Mission® esiRNA targeting human AAMP (esiRNA1) were purchased from Sigma-Aldrich (Missouri, USA). Mission® esiRNA are a heterogeneous mixture of short interfering RNAs (siRNAs) that all target the same mRNA sequence, which conduct highly specific and effective gene silencing. The negative control siRNA was from Eurogentec (SR-CL000-005). Transfections were performed as previously described using 10 nM of siRNAs and, after 48 h, the transfected cells were evaluated.

### Wound healing and invasion chamber assays

Wound healing assays were performed in 6-well plates covered by confluent monolayer of A549, NCI-H460, or MRC5 cells [treated and untreated with ang-(1–7) or miRNA-transfected cell groups, mimic or inhibitor]. For each assay, a scratch was made with a sterile 200 μl pipet tip, which scrapped off the cells, creating a visible line at the wound edge. Cells were carefully washed with PBS to remove cellular debris and fresh culture medium was replaced. Immediately, after the wound (0 h time-point) and after 24 hours, cells were analyzed and photographed under phase-contrast microscopy to monitor the movement of the cells into the wound. The assays were performed several times, and the measurements were performed in triplicate; mean values of consecutive tracings were computed and expressed as percentage of closure from the original wound. The wound-healing curve was obtained using Graphpad Prism version 5.

For the invasion chamber assay, 24-well Corning® BioCoat™ Matrigel® Invasion Chamber with 8.0 micron pore size polyester (PET) membrane was used. A total of (2.0 × 10^4^) cells were suspended in a medium containing 0.4% of serum and seeded into the upper wells, following the instructions of the supplier of the chambers. After 24 h incubation at 37°C and in an atmosphere that contained 5% CO_2_, cells that invaded through the Matrigel (those that migrated to the lower chamber during incubation) were fixed and stained with 100% methanol and 1% toluidine blue respectively, for 2 min in each solution. After extensively washing in PBS, the inserts were air dried, and the number of cells that migrated to the bottom of the chamber was photographed and counted using a phase-contrast microscopy on ten randomly selected fields. Three sets of experiments were carried out.

### Protein analysis

Whole cell extracts were prepared as previously described [22]. Equal amounts of protein (50 μg) of each investigated condition were separated by electrophoresis in 10% polyacrylamide gels and then electrotransferred to polyvinylidene fluoride (PVDF) membranes. The membranes were immunoblotted overnight with rabbit anti-angio associated migratory cell protein (AAMP) (ABCAM, USA), or rabbit anti-β actin (Cell Signaling, USA) polyclonal antibodies, followed by 2 h of incubation with a horseradish peroxidase-conjugated goat anti-rabbit antibody (Cayman Chemical). Immunoreactive bands were visualized using a chemiluminescent detection kit (ECL^TM^, GE Healthcare) and exposed to Hyperfilm (GE Healthcare). The bands were quantified with Quantity One Software, Biorad.

### Reporter assay

A549 cell were plated at a density of 6 x 10^4^ cells into 24-well plates 24 h before transfection and transiently transfected with 100 ng of the pGL3-Control vector (Promega), or 100 ng of pGL3-AAMP-3’-UTR (pGL3-Control vector containing the cDNA sequence of the 3’ untranslated sequence, UTR, of the *AAMP* gene, 182–201 bp, 5’-GCCCTCCCACCCTTGACCAGAC), or pGL3-AAMP mut-3’-UTR (pGL3-Control vector containing mutated cDNA sequence of the 3’- UTR, of the *AAMP* gene, 182–201 bp, 5’-GCAAUAACACAAUUGUCAGGAC). In addition, cells were cotransfected with 30 nM of custom designed miR-149-3p mimic or inhibitor (mirVana™miRNA inhibitor, Thermo Fisher Scientific). Transfections were performed using Lipofectamine® 2000 Transfection Reagent (Thermo Fisher Scientific), as previously described. The Renilla luciferase reporter plasmid (pRL-TK) was used as the internal control for the transfection efficiency. The assays were performed in triplicate, and fold changes were calculated using values normalized to Renilla luciferase activity. The luciferase activities were measured using a TD20/20 luminometer (Turner Designs).

### Graphs and statistical analyses

Values from three independent assays were used for the analyses, and graphs were generated using Graph Pad Prism^®^ 5. The differences between the control, treated and miRNA mimics or inhibitors transfected groups were also measured using one-way analysis of variance (ANOVA), followed by Dunnett’s test. Significance was set at p < 0.05.

## Results

### MicroRNA signatures and correlation with the angiotensin-(1–7) treatment

**Fig 1:**
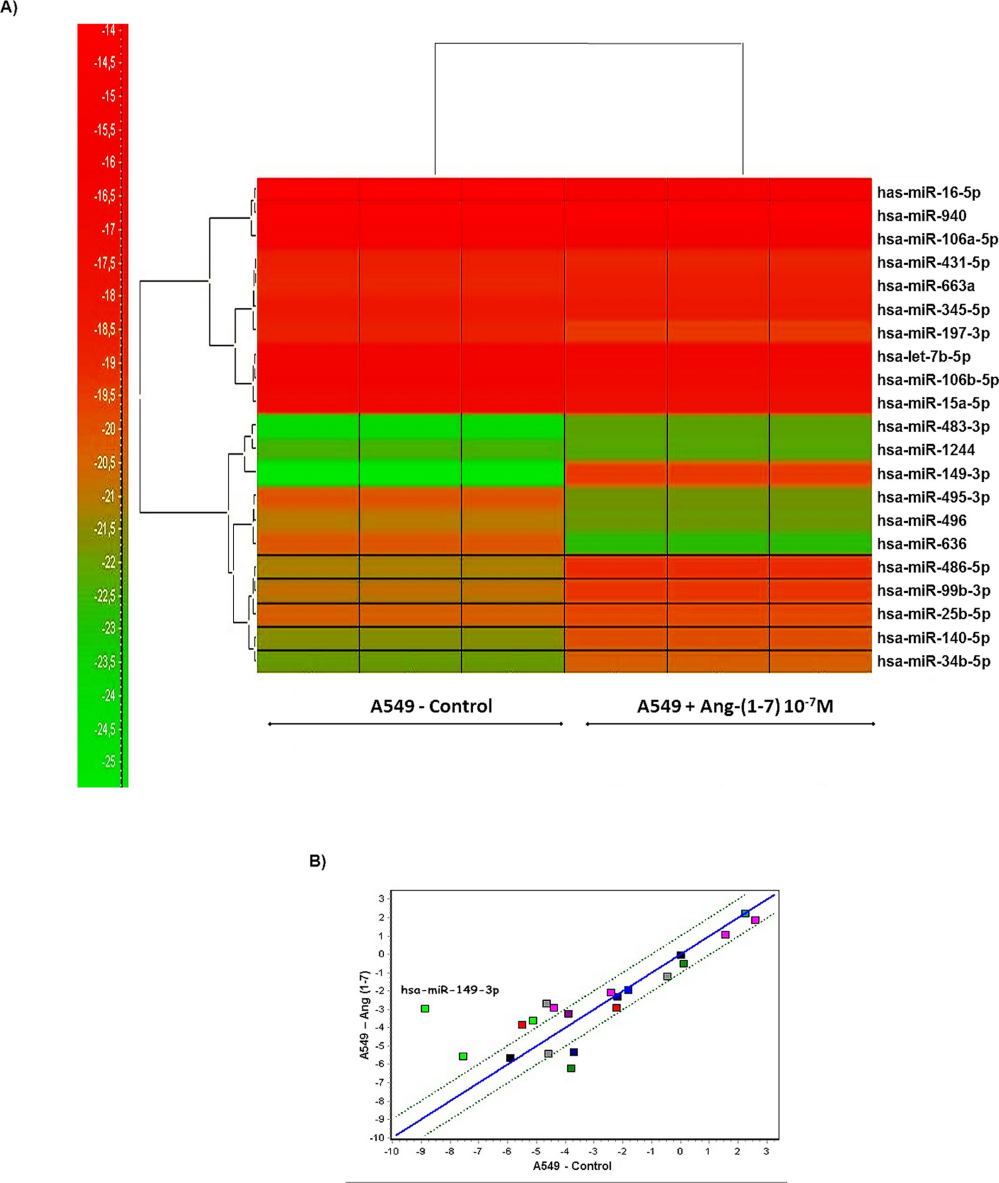
miRNA expression profiles in angiotensin-(1–7)-treated or untreated A549 cells. A) Heatmap of the 21 miRNAs with altered expression levels after cellular treatment with the heptapeptide. Each column represents an individual cell culture and each row represents an individual miRNA. Colors of the heatmap represent the Z-score: higher–red, lower–green. B) Correlation of expression profiles with significantly different miRNA expression after the heptapeptide treatment. The x and the y intercepts represent relative expression values of A549 untreated and ang-(1–7)-treated cells, respectively. The graphs were packed using GeneEX (Exiquon, Denmark) software and significance was set at p ≤ 0.001

To address relevant molecules that modulate tumor growth mediated by the effect of the heptapeptide, miRNA expression profiles of ang-(1–7)-treated and untreated pulmonary cancer cells were obtained. In total, 21 miRNAs were differentially expressed between the cellular groups (p ≤ 0.001). Fig 1A presents the heatmap for the microarray data describing the expression profile of miRNA in A549 untreated cells (control condition) x ang-(1–7)-treated cells. Two main clusters of miRNA expression dependent on the cellular treatment were observed, and Table 2 lists all miRNAs for which the expression levels differ between the investigated conditions. The largest increases in expression occurred in miR-149-3p, miR-486-5p, miR-483-3p, miR-34b-5p, miR-99b-3p and miR-140-5p at 5.925, 1.968, 1.965, 1.628, 1.503 and 1.488 fold of the control level, respectively. The largest decreases in expression occurred in miR-636 and miR-495-3p at 2.423 and 1.62 fold of the control level, respectively. The statistic correlation analyses based on the miRNA profiles of the investigated cells are presented in a scatter plot, along with the miRNA distribution (Fig 1B). The graph indicates that changes are more prominent in miRNA-149-3p expression pattern when cells were treated with the heptapeptide. Therefore, based on the mathematical analysis, the combined results directed our analyses to the functional investigation of the miRNA-149-3p.

**Table 2:** List of miRNAs differentially expressed between ang-(1–7) treated and untreated cells (P value: ≤ 0.001).

miRNAs	Fold change log scale
hsa-miR-149-3p	5,92533
hsa-miR-486-5p	1,96833
hsa-miR-483-3p	1,96533
hsa-miR-34b-5p	1,62833
hsa-miR-99b-3p	1,50333
hsa-miR-140-5p	1,48833
hsa-miR-25-5p	0,64033
hsa-miR-663a	0,30133
hsa-miR-1244	0,23733
hsa-miR-940	0,01433
hsa-let-7b-5p	-0,05967
hsa-miR-345-5p	-0,11067
hsa-miR-431-5p	-0,14667
hsa-miR-106a-5p	-0,45867
hsa-miR-106b-5p	-0,60267
hsa-miR-197-3p	-0,69067
hsa-miR-16-5p	-0,73167
hsa-miR-15a-5p	-0,74667
hsa-miR-496	-0,84067
hsa-miR-495-3p	-1,62267
hsa-miR-636	-2,42367

**Fig 2:**
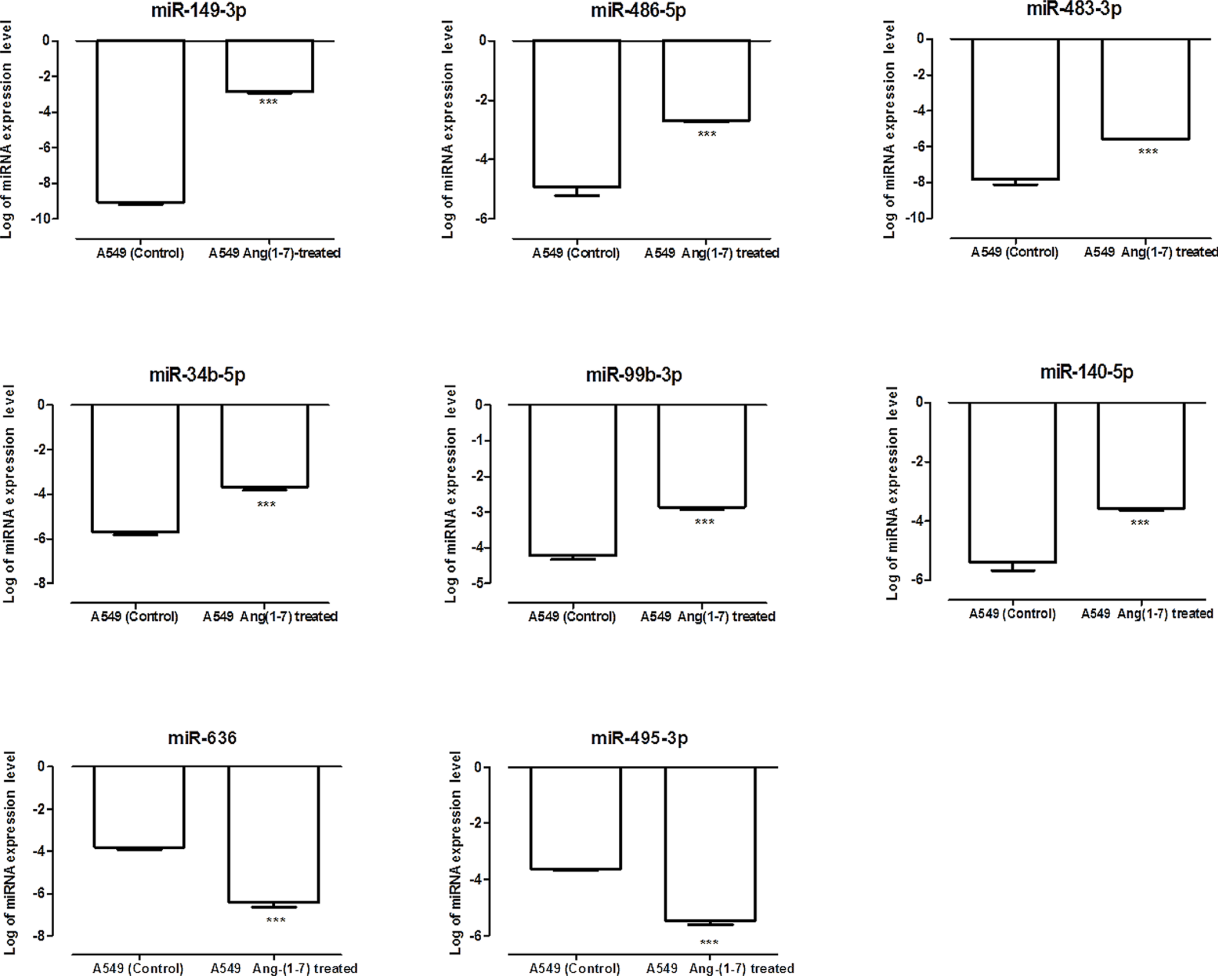
Expression levels of selected miRNAs in angiotensin-(1–7)-treated or untreated A549 cells. Relative miRNA expression in logarithmic scale by qRT-PCR corroborated the results found in miRNA PCR arrays. The graphs present values from the average from three independent experiments, and the error bars represent the standard deviation of the mean. ANOVA testing showed significant differences between the A549 untreated culture (control) and the ang-(1–7)-treated cells. The significance level was set at p<0.05 (***).

To validate the results from miRCURY LNA™ PCR array, isolated qRT-PCR miRNAs assays were performed for the eight miRNAs, whose expression level changed considerable after ang-(1–7) treatment. Fig 2 presents the results for the miRNA expression pattern, which follows the same results found in the PCR array.

### Modulatory effect of angiotensin-(1–7) and the miRNA-149-3p in morphofunctional aspects of cell migration

**Fig 3:**
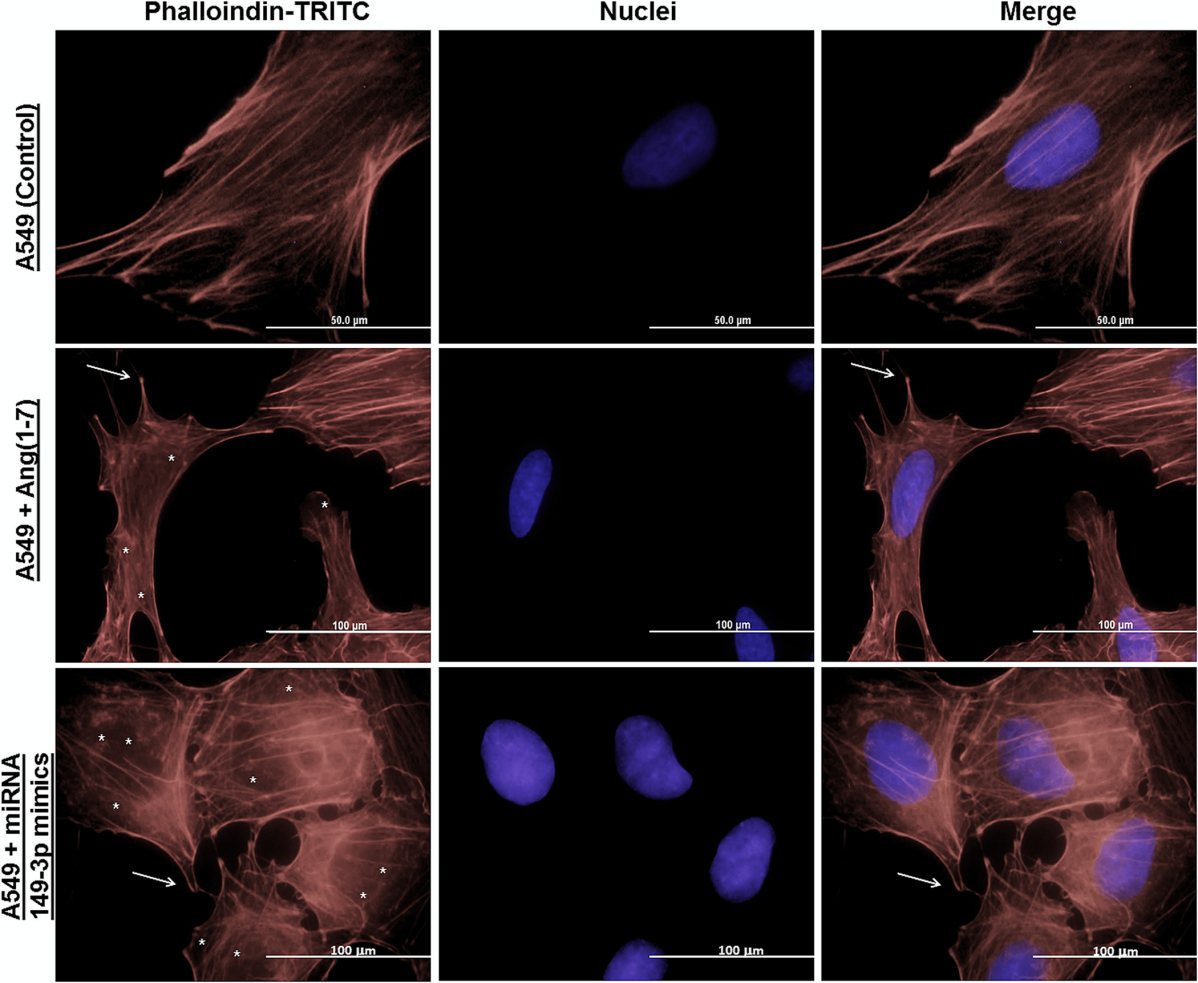
Immunocytochemistry of actin filaments of A549 cells. Different groups of A549 cells were grown on coverslips and then stained with phalloidin-TRITC and DAPI (nuclei) to investigate morphologic changes in cellular groups. Arrows (→) indicate filopodia and asterisks (*) indicate actin filaments disassemble in the images. Scale bar are assigned.

In order to investigate the modulatory effect of the ang-(1–7)-treatment in cellular migratory process, we started our analyses by investigating the distribution of actin filaments, which helps to infer cellular morphological changes. Fig 3 presents the results observed in untreated A549 (control condition) and after cellular treatment with either the heptapeptide or the miRNA mimics. In the control group, a very well organized distribution of filamentous (F) actin was found. When compared to the control, both the treated or transfected cells showed an increased percentage of disassembled actin filaments and the presence of filopodia were observed. Especially, in miRNA mimics transfected cells, the disassemble level of actin filaments is prominent. Analyses for 149-3p miRNA inhibitor were also performed and no significant changes were observed compared to the A549 control condition (data not shown). Taken together, these results indicate that the peptide is able to modulate the organization and assembling of actin fliaments in a cell and the miRNA-149-3p acts as a relevant molecule in this remodeling process. It is well described that actin participates in many important cellular events besides cell motility and maintenance of cell shape [23,24,25]. Actin contributes to cell signaling, intracellular transport of organelles and vesicles [26,27,28], wound healing [23], cancer cell metastasis [25, 29] and even the control of gene expression [30,31]. The results in the fluorescence microscopy analyses reinforce the observation that the heptapeptide has the ability to modulate cellular migration.

**Fig 4:**
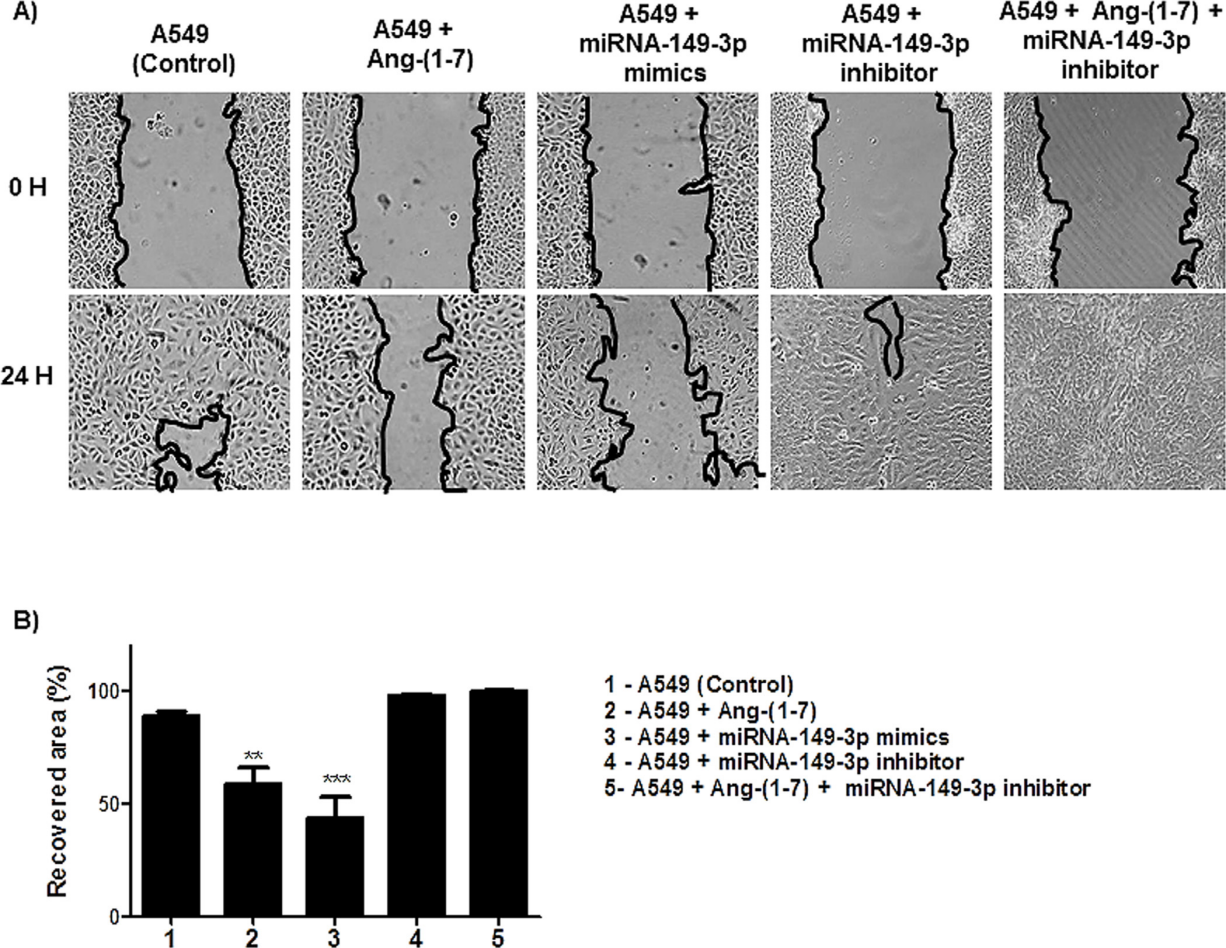
Angiotensin—(1–7) and miRNA-149-3p affects the motility and migration of A549 cells. A) Different groups of A549 cells were grown in 6-well plates. Under confluency, cellular migration was examined by wound healing assay and the recovery area of the injury was measured. B) The graph presents values of the average from three independent experiments, and the error bars represent the standard deviation of the mean. ANOVA testing showed significant differences in the ability to recover the injured are of the culture between A549 untreated culture (control) and the cellular groups treated with ang-(1–7) or transfected with miRNA-149-3p mimics. The significance level was set at p<0.05 (***).

Next, we analyzed the effect of the ang-(1–7) and the miRNA-149-3p in the migratory processes of lung tumor cells. Wound healing and invasion chamber assays were performed using three different lung cells lines. The assays revealed that the heptapeptide and the mimic reduced the migration of A549 cells (Fig 4). After 24 h of the initial culture injury, approximately 88.8% of the surface area was recovered in A549 control culture. In contrast, in the heptapeptide-treated cultures, the recovery area was nearly 58.57%; in the miRNA-mimic transfect cells the recovered area of the culture was even lower (43.68%). As observed in miRNA-inhibitor transfected cell culture, the recovery area reached almost complete confluency (98.01%). In addition, a rescue experiment was performed by knocking down miRNA-149-3p in ang-(1–7) treated cells and the results demonstrated a complete recovery of the injured area. The combined results demonstrate the cellular physiologic effect of the miRNAs in the migratory processes of A549 cell line.

**Fig 5:**
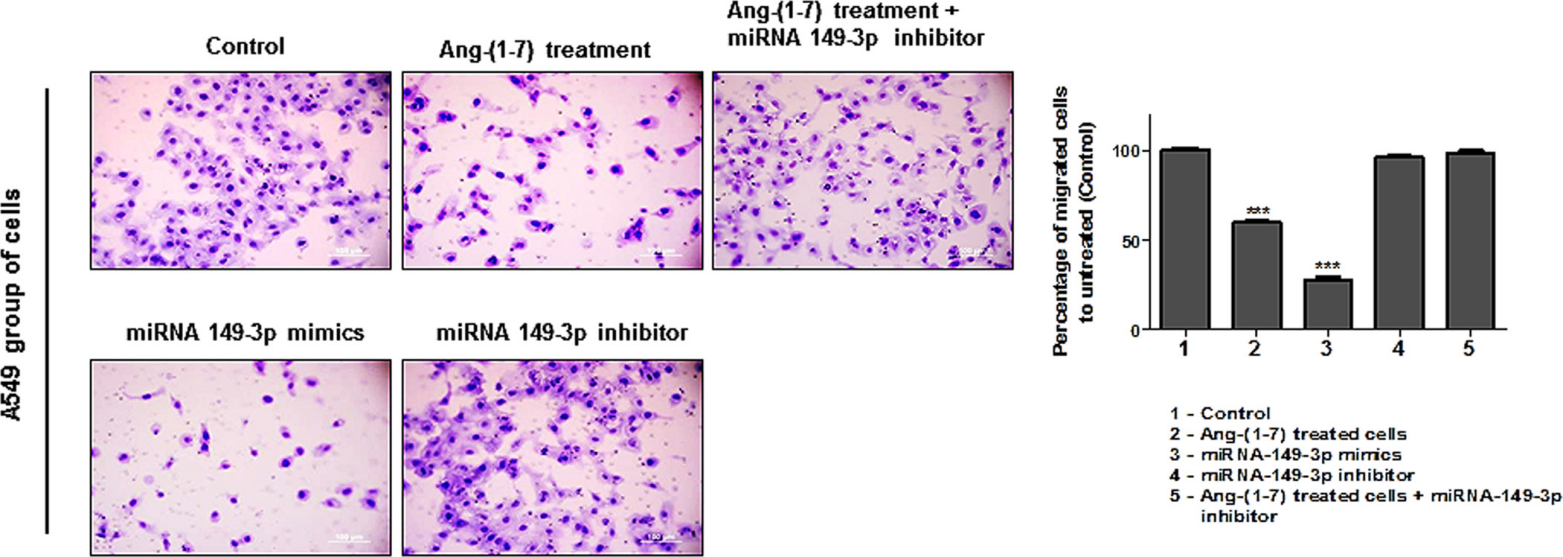
Angiotensin—(1–7) and miRNA-149-3p repress A549 cell invasion. Transwell chamber invasion assays of A549 cells either treated or left untreated with the heptapeptide, or transfected with miRNA-149-3p mimics or inhibitor were performed and representative fields of invaded and stained cells are shown (left). The graph presents values of the average from three independent experiments, and the error bars represent the standard deviation of the mean. ANOVA testing showed significant differences between the ability of A549 untreated culture (control) to invade the chambers and the cellular groups treated with ang-(1–7) or transfected with miR-149-3p mimics. The significance level was set at p<0.05 (***).

To validate the effect of the angiotensin-(1–7) and the miRNA-mimic on the migration process, invasion chamber assays were performed for each independent group of A549. Fig 5 presents the results and shows that when cells were treated with either ang-(1–7) or miRNA-149-3p mimic, the cellular invasion processes were reduced (Fig 5). Compared to the untreated group (control), the number of cells that invaded the bottom of the chamber in the ang-(1–7) treated cells was reduced by approximately 39.77%. The ability of miRNA-149-3p mimic transfected cells reach the bottom of the chamber was even lower; the ability of transfected cell migration reduced 72%. Otherwise, cellular transfection with miRNA-149-3p inhibitor did not change migratory capacity of the cells through the chamber, as was observed in the rescue experiment [miRNA-149-3p inhibitor transfection in ang-(1–7) treated cells], when compared to the cellular migratory pattern of the control group.

**Fig 6:**
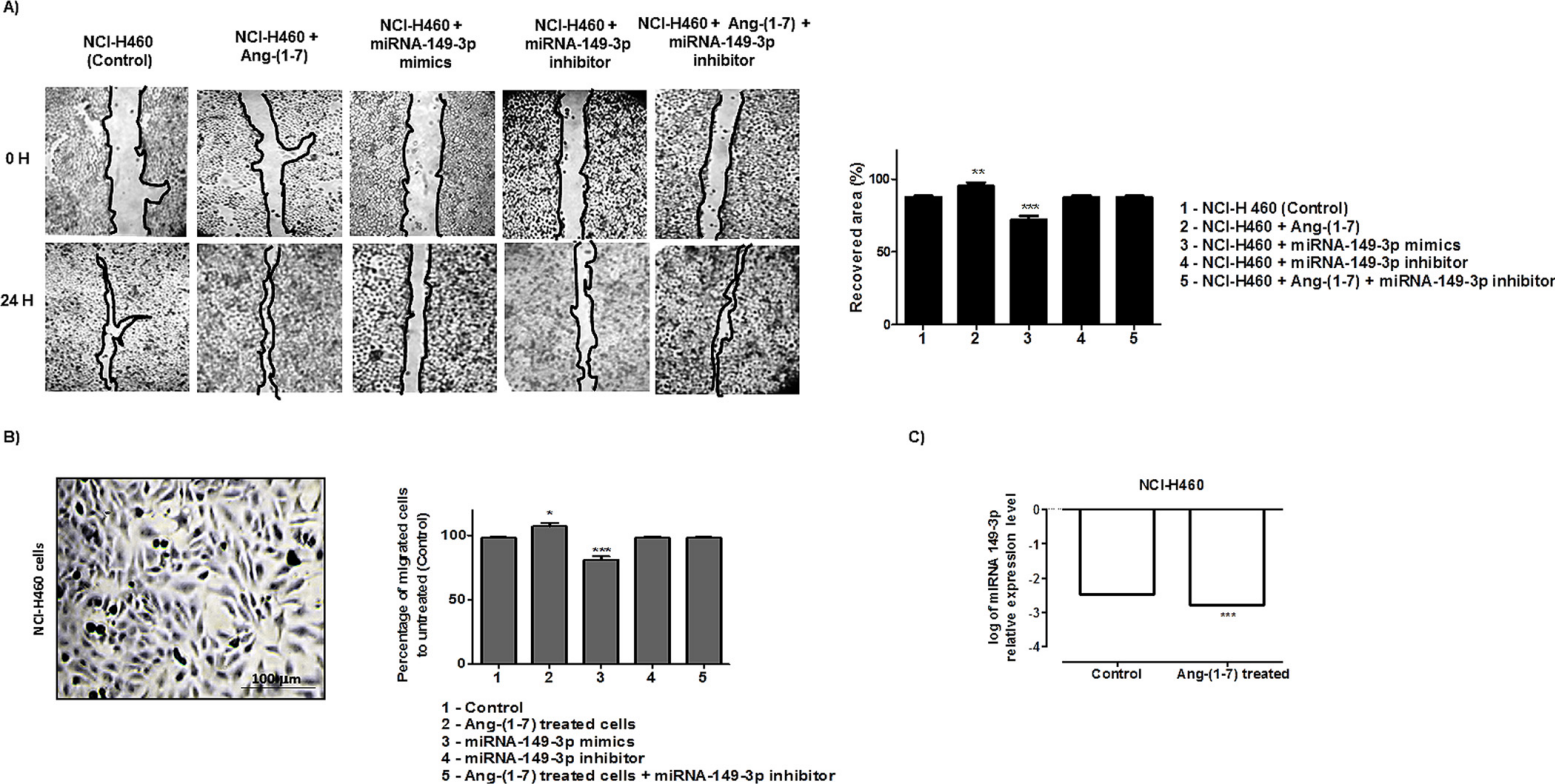
Angiotensin—(1–7) and miRNA-149-3p modulates NCI-H460 cell motility and migration. A) NCI-H460 cells were grown in 6-well plates under different culture conditions. Under confluency, cellular migration was verified by wound healing assay and the recovery area of the injury was measured in each condition analyzed and plotted in graphs. B) Transwell chamber invasion assays of NCI-H460 cells either treated or untreated with the heptapeptide, or transfected with miRNA-149-3p mimics or inhibitor, or in cells cultured in the presence of the ang-(1–7) with subsequent miRNA-149-3p knocked down. Representative field of invaded and stained cells is shown (left), and the measurements were plotted in graphs. In the assays represented in A and B, ANOVA testing showed significant differences between the ability of NCI-H460 cells to recover the injured area of the culture or migrates through the chambers in ang-(1–7) or transfected with miRNA-149-3p mimics groups, compared to the untreated culture (control). C) Relative miRNA expression in logarithmic scale by qRT-PCR. All graphs presents values of the average from three independent experiments, and the error bars represent the standard deviation of the mean. The significance level was set at p<0.05 (***).

Moreover, the wound healing assays performed using NCI-H460 (Fig 6A) demonstrated nearly 86.6% recovery in injured area of the control culture, after 24 h of culture scratch. In the heptapeptide-treated cultures, the recovery area was approximately 95.2% and in miRNA-mimic transfect cells, the recovery was nearly 72% of totally injured area. The other two tested culture conditions demonstrated 86% average recovery of the wound, which is similar to the results observed in control groups. The invasion chamber assays performed for each independent group of NCI-H460 (Fig 6B) demonstrated that when compared to the untreated group (control), the number of cells that invaded the bottom of the chamber in the ang-(1–7) treated cells increased by approximately 13%. The miRNA-mimic transfect cells had a 17% reduction in migration, compared to the rates found in control group. However, in the miRNA-149-3p inhibitor transfected cells group and in the ang-(1–7) treated cells, were the miRNA-149-3p was knocked down, the pattern of cellular chambers invasion was similar to the control group. Together the results also demonstrated cellular physiologic effect of the miRNAs in the migratory processes of NCI-H460 cell line. The difference observed in healing and migration pattern of this cellular group, when compared to the A549 cells, is possibly connected to the miRNA 149-3p expression pattern. Fig 6C demonstrated a significant reduction in 149-3p miRNA level in NCI-H460 heptapeptide treated cells (-0.309).

**Fig 7:**
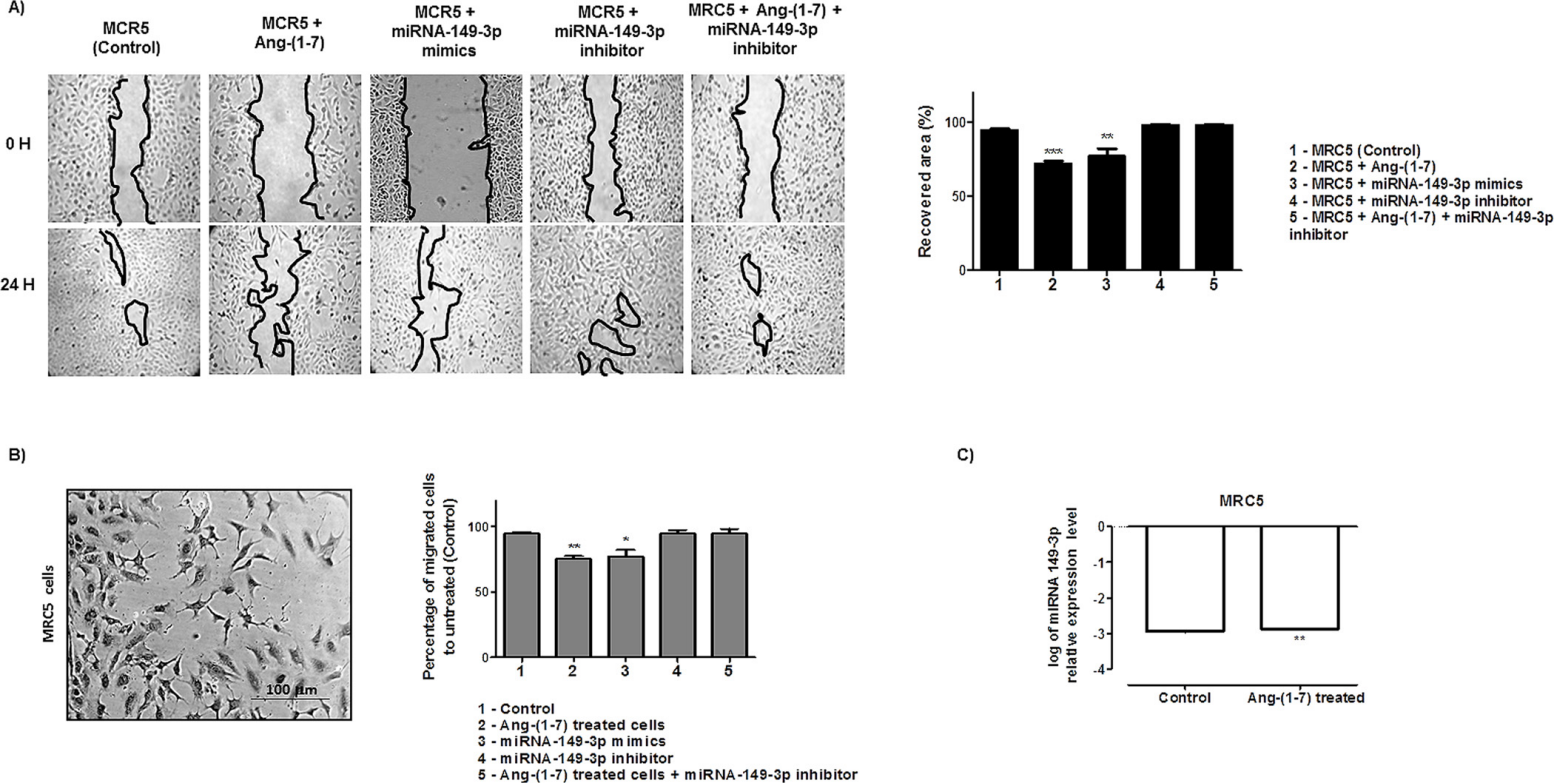
Angiotensin—(1–7) and miRNA-149-3p modulates MRC-5 cell motility and migration. A) MRC-5 cells were grown in 6-well plates under different culture conditions. Under confluency, cellular migration was verified by wound healing assay and the recovery area of the injury was measured in each condition analyzed and plotted in graphs. B) Transwell chamber invasion assays of MRC-5 cells either treated or untreated with the heptapeptide, or transfected with miRNA-149-3p mimics or inhibitor, or in cells cultured in the presence of the ang-(1–7) with subsequent miRNA-149-3p knocked down. Representative field of invaded and stained cells is shown (left) and the measurements were plotted in graphs. In the assays represented in A and B, ANOVA testing showed significant differences between the ability of MRC-5 cells to recover the injured area of the culture or migrates through the chambers in ang-(1–7) or transfected with miRNA-149-3p mimics groups, compared to the untreated culture (control). C) Relative miRNA expression in logarithmic scale by qRT-PCR. All graphs presents values of the average from three independent experiments, and the error bars represent the standard deviation of the mean. The significance level was set at p<0.05 (***).

The same analyses were accomplished for MRC5 cell line. The wound healing assays presented in Fig 7A demonstrated nearly 94.6% recovery in injured area of the control culture, after 24 h of culture scratch. In the heptapeptide-treated cultures, the recovery area was approximately 72% and in miRNA-mimic transfect cells, the recovery was nearly 77% of totally injured area. The other two culture conditions demonstrated 98% average recovery of the wounds. The invasion chamber assays performed for each independent group of MRC-5 cells (Fig 7B) demonstrated that when compared to the untreated group (control), the number of cells that invaded the bottom of the chamber in the ang-(1–7) treated cells decreased by approximately 20%. In miRNA-mimic transfect cells, an 18.6% reduction in migration was observed. In addition, the last two investigated groups [miRNA-149-3p inhibitor transfected cells and ang-(1–7)-treated cells followed by the miRNA-149-3p knocking down–rescued group] present a migratory pattern similar to the control group. Hence, the results also emphasize the molecular connections of the miRNA-149-3p with the cellular migratory processes, whose pattern of healing and migration resembles that found in A549 cells. In addition, a significant increase in 149-3p miRNA level was found in MRC5 heptapeptide treated cells (0.06696 –Fig 7C), which could support the results found.

Moreover, despite the fact that each cell line has its particular physiological behavior, considering the differences in molecular and biochemical connections that play a relevant function in cellular homeostasis, the miRNA-149-3p can be appointed as an important element in controlling migratory cellular processes.

### miRNA-149-3p and it modulatory effect in predicted target genes

**Fig 8:**
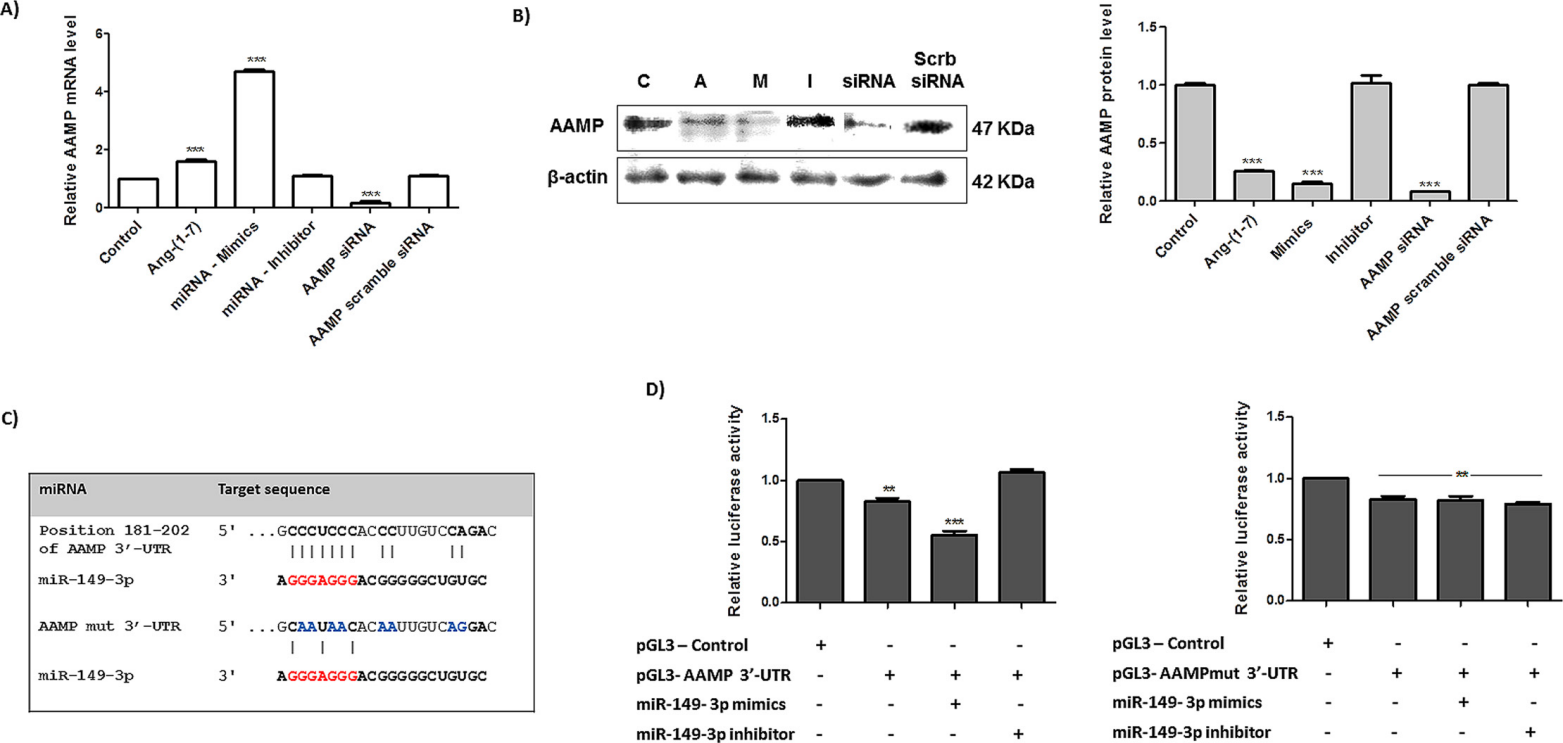
miRNA-149-3p modulates AAMP expression by miRNA-mRNA physical interaction. The expression level of AAMP in A549 cells either treated or untreated with the heptapeptide angiotensin-(1–7), or transfected with miRNA-149-3p mimic or inhibitor are presented. In addition, the effect of AAMP siRNA were also investigated. A) Relative mRNA level of AAMP in different groups of cell investigated. B) Protein extracts from different groups of cells were analyzed using Western blots and specific antibodies. β-actin was used as a loading control. C) Schematic representation of AAMP and AAMP mutated mRNA target site and the miRNA-149-3p. D) Relative luciferase activity in cells transfected with pGL3 plasmids (Control, AAMP-3’-UTR and AAMPmut-3’-UTR) and miRNA-149-3p mimics or inhibitor; the results were plotted in representative graphs. ANOVA analysis found significant differences between the control and cell samples; the significance level was set at p <0.05 (***).

Based on statistic correlation analyses of the miRNA arrays and their potential ability to modulate cellular migratory processes, we selected the miRNA-149-3p for functional studies. We evaluated its modulatory function in migration process and how it connects to the physiological effect of the ang-(1–7) in the control of tumor cell growth and migration. To verify the effect of the heptapeptide and the miRNA 149-3p in the selected 26 potential target genes that are correlated with the biological process of migration (Table 1), A549 cells were treated or untreated with ang-(1–7), and transfected with either the miRNA mimic or inhibitor. Next, qPCRs were performed using sets of specific oligonucleotides (sequence not shown) and the analyses demonstrated that the expression level of 20 out of 26 genes (CADM1, CADM3, CLDN10, COL1A1, ELN, ITGA1, ITGA2, ITGA3, ITGA5, LAMC1, LAMC3, MMP2, MMP15, PCDHGB5, PCDHGB7, SMAD2, SMAD3, TIMP2, TIMP3 and TJAP1) did not change in the investigated groups of cells. The analyses also revealed that in the A549 cells transfected with either the 149-3p miRNA mimic or miRNA inhibitor did not change the mRNA expression level of ADAM11, COL1A2, MMP14, PCDHGA10 and PCDHGC13, when compared to the control cell culture (data not shown). It is worth mentioning that Lipofectamine® 2000 did not change the expression pattern of investigated genes compared to the pattern observed in the control group. Moreover, an interesting pattern of expression was observed for the gene AAMP, which has inverse correlation with the expression level of AAMP protein. Fig 8 presents the relative gene expression and protein level for the AAMP molecule in different groups of A549 cells under investigation. As shown in A, cellular treatment with the heptapeptide increased the relative mRNA level of AAMP in approximately 61%, and cellular transfection with the miRNA-149-3p mimics induced super expression of the investigated mRNA (~480%), when compared with the pattern of expression in the untreated group of cells. Otherwise, the transfection with miRNA-149-3p inhibitor did not change the relative mRNA level of AAMP, when compared to the results found in untreated cell cultures (control condition). In addition, no major changes in AAMP expression level were observed in cell cultures exclusively incubated with the lipofectamine, when compared to the expression found in control cultures (data not shown).

Next, we tested whether the AAMP protein level changed in different groups of A549 cells. Fig 8B shows the results of this experiment. In contrast with the mRNA level, the protein level in ang-(1–7) treated cells decreased by approximately 73.8% when compared to the untreated group, and in the miRNA-149-3p mimic-transfected cells an even lower level of protein was observed (84.3% lower than the control condition). The miRNA-inhibitor transfection did not change AAMP molecule when compared to the protein level of the control group of cells. Taken together the results of the above experiments strongly suggest a direct modulatory effect of the miRNA in mediating AAMP protein expression. This observation is largely supported by previous studies, which demonstrates that translational repression is the primary event by which miRNAs repress gene expression, considering the high frequency of no perfect miRNA-mRNA complementary [32–36]. In ang-(1–7) treated cells, the high expression level of miRNA-149-3p support posttranscriptional repression of AAMP, by blocking the protein translation. In addition, AAMP siRNA assay was performed to verify the mechanistic approach of AAMP silencing. During canonical RNA interference (RNAi) pathway, RNA molecules are degraded; however, in some circumstances, silencing can involve either translational repression or exonucleolytic degradation [33]. In our model, the esiRNA1 mediated the classical mechanism of silencing by degrading the AAMP mRNA, which results in low level of AAMP protein and the result is clearly observed in Fig 8A and 8B. Moreover, despite the differences in the mechanistic approaches of AAMP protein knockdown by either the miRNA-mimics 149-3p or the AMMP esiRNA, they considerably reduced cellular protein level. In A549 transfected-cells with the two different molecules, the same cellular physiological behavior in wound healing and migration chamber assay was observed (S2 Fig), reemphasizing the relevance of the protein to tumor cell control.

To confirm if AAMP 3’-UTR has a true target site of miRNA-1493p, dual-luciferase assays were performed to verify physical interaction between the putative target sequence in the human AAMP mRNA and the investigated miRNA (Fig 8C and 8D). The potential target site of miRNA-149-3p was cloned into pGL3 plasmid, and this clone was cotransfected into A549 cells along with miRNA-149-3p mimic or inhibitor. The *Renilla* luciferase vector (pRL-TK) was used to normalize the assays and the pGL3-Control vector was used to normalize the luciferase production by the cells in the presence of an empty vector. As is shown in Fig 8D, the AAMP-3’- UTR construct transfected cells reduced the production of luciferase by approximately 17% when compared to the cells transfected with the pGL3- Control vector. Moreover, in the presence of miRNA mimics, the luciferase activity produced in co-transfected A549 was even lower: a 44.4% reduction in luciferase production was verified when compared to the pGL3-Control group. Additionally, the presence of the specific miRNA 149-3p inhibitor in transfected cells did not result in major changes in the luciferase production by AAMP-3’- UTR construct when compared to the control group of cells, which reinforce the observation that this specific inhibitor blocks the natural miRNA binding to the 3’-UTR of AAMP, facilitating extra rounds of luciferase translation. In addition, luciferase assays were performed using AAMPmut-3’- UTR construct and Fig 8D also present the results. The presence of the plasmid pGL3- AAMPmut-3’- UTR reduced the luciferase expression by 18% when compared to the production of the luciferase by A549 transfected cells with the pGL3-Control vector. The assays performed in the presence of either miRNA-mimic or inhibitor did not affected the production of luciferase, reinforcing the specificity of the physical interaction between 3’-UTR of AAMP and the miRNA-149-3p sequence.

Therefore, we conclude that the miRNA-149-3p is a relevant molecule, which is modulated by the heptapeptide and plays a relevant function in mechanisms that control migratory rates of cells in the context of the system biology in each individual lung cell line.

## Discussion

Lung cancer is one of the most frequent types of cancer in humans and a leading cause of death worldwide. Thus, the development of an effective treatment is needed. In more recently years, miRNAs has been appointed as relevant molecules that play major functions in the progression and/ or control of several different diseases, including cancer. Such observations have conducted researches on functional aspects of miRNAs, which could be useful for the development of new therapeutic strategies. Furthermore, the heptapeptide angiotensin-(1–7) has demonstrated the ability to control the growth rates of human lung cancer cells in vitro, and in vivo [7,8,9] and may be consider as a promising cancer treatment. Thus, our study investigated the modulatory effect of the heptapeptide on the expression pattern of miRNAs in lung tumor cells, to characterize relevant elements that modulate tumoral cell behavior.

In this study, twenty one miRNAs presented significant statistic differences between the investigated A549 groups of cells and more prominent changes were observed in miRNA-149-3p expression when cells were treated with the heptapeptide (Fig 1), which could indicate a modulatory event in the control of tumor migration. Long et al. (2013) [37] reported a decreased level of this miRNA in chordomas, which could be correlated with tumor aggressiveness and invasion. This is a rare slow-growing neoplasm [38] that arises from remnant structures of the notochord; however, it has high metastatic potential [39]. Therefore, in our study, the increased level of miRNA-149-3p in A549 cells, after the heptapeptide treatment, supports reduced rates of cellular migration. In addition, the analyses of the other two different cells lines (NCI-H460 and MRC5) reinforce the observation that the expression level of miRNA-149-3p is able to modulate cellular migration. In lung tumor cells NCI-H460, the peptide considerably reduced the expression level of the investigated miRNA, which accelerates the growth and migration rates. However, in normal lung cell line MRC5, a significant increase in the expression level of miRNA-149-3p was observed after ang-(1–7) treatment, which matches with the decreased pattern of cell growth migration observed in wound healing and transwell assays. The combined results reinforce the relevant function of the miRNA-149-3p in the controll of migratory cellular processes.

It is likely that miRNAs are not the only element that could contribute to the control of cancer cell migration and metastasis; however, they have their own signature in either physiological or pathological condition. Therefore, the understanding of the transcription processes of such molecules and its regulation in pathologies will help to access the development of new therapeutic approaches. Unfortunately, despite all the scientific efforts, the mechanistic pathways and elements that control cellular expression of miRNA have still not been clarified. This is a result of poor promoter sequence characterization [40]. Researches in this area revealed that miRNAs have their own transcription initiation regions, which are found either in the intergenic or intronic region, adding new layers of complexity to the regulation of miRNA expression [40,41]. In the present work, under the influence of ang-(1–7), each lung cell line activates particular molecular routes that influence the expression level of the miRNA-149-3p, which modulates cellular migration processes. Hence, the positive or the negative expression of the investigated miRNA and their cellular physiological behavior presented above should be consider as an interesting molecule for future investigation of alternative therapies for the treatment of lung cancer.

To reinforce the cellular analyses of the effect of either ang-(1–7) or higher level of miRNA-149-3p mimics, we used A549 cells as our model. When compared to the control culture, the presence of the heptapeptide induces disassembly of a considerable number of actin F filaments in most of the cells under the treatment; on the other hand, cellular structures that resemble filopodia were observed (Fig 3). The combined characteristics in treated lung cell are probably modulating the migration rates. Meanwhile, the presence of higher level of miRNA-149-3p mimics also induces higher level of actin F depolymerization. Discrete filopodia were also observed in transfected cells, suggesting that the combination of a more disorganized actin distribution and the presence of discret filopodia physically contribute to the reduction of cellular migration, as was verified in wound healing and membrane transwell assays. This observation reemphasizes the molecular connections with the miR-149-3p and cellular migratory processes. Moreover, such conformational rearrangements in cell morphology are probably correlated with changes in the pattern of GTPase and kinases function. Edelstein-Keshet (2016)[42] and Nobes and Hall (1995)[43] reinforce the relevance of Rac, Rho and small GTPase in actin assembly and lamellipodia/ filopodiaformation. Furthermore, it has been well described that ang-(1–7) is able to modulate the AKT-PKB pathways in cardiac cells and other tissues [44–47]. In our assays, miRNA-149-3p mimics were also able to modulate the expression level of AKT in the three different cell lines (data not shown), indicating its direct correlation with morphological changes observed in microscopy analyses, which influence the migratory processes. Furthermore, the wound healing and the invasion chamber assays clearly demonstrated the modulatory effect of the miRNA-149-3p in cellular migration (Figs 4, 5, 6 and 7). It was well described that the healing process of the injured site is an extremely complex process, which involves several cellular events, and, during this process, cells migrate to cover the injured surface [48]. In cancer cells, migratory and invasive characteristics of the cells are correlated with the aggressiveness of the disease. The modulatory effect of the angiotensin-(1–7) and the investigated miRNA in controlling the processes of cancer cell migration and invasiveness of chambers reinforces the characteristic of these small molecules in modulating such pathological mechanisms in cell culture. Therefore, our findings reinforce the hypothesis that the miRNA149-3p controls a network of cellular signaling as does angiotensin-(1–7), which modulates cellular migration processes. The identification and characterization of cellular function of the miRNA-149-3p in lung tumor cells should be consider a jackpot element for future drug development.

To validate our hypothesis and explore the mechanisms by which the angiotensin-(1–7) controls cellular tumor migration and the relevance of miRNA-149-3p in this cellular process, we used bioinformatics tools to direct our investigation. The selected messenger molecules, containing potential miRNA target sites, were correlated with migration and/ or adhesion mechanisms. This could help us to construct a cellular signaling network in tumor cells treated with the heptapeptide or presenting higher level of the miRNA 149-3p. Once again, using A549 as our cellular model, AAMP molecule presented significant changes in its level in either ang-(1–7) treated culture or after miRNA-mimic cellular transfection, when compared to the level found in control cellular culture (Fig 8). Moreover, the molecular pattern of AAMP gene expression demonstrated an inversed correlation with its protein level, which reinforces the posttranscriptional repression of the protein. Such observation is corroborated by the AAMP siRNA analyses, considering that such molecules mediate classical mechanisms of RNAi, which conducts RNA molecules degradation.

AAMP or angio-associated migratory cell protein is an element directly associated with angiogenesis and migration of endothelial cells, including cancer cells [49]. In smooth muscle cell, AAMP acts in cellular migration via the RhoA pathways, which reinforces the cytoskeleton remodeling during migration processes and controls cell migration [50]. In addition, the widespread localization of AAMP transcripts and protein [49] facilitates its interconnections with several biological mechanisms in a cell, and it was found to be expressed in several tumor tissues [51]. Our current study demonstrated that the effect of the heptapeptide in controlling tumor cell migration is cellular lineage dependent and correlated with the expression level of miRNA-149-3p mimics. Furthermore, the physical interaction between AAMP transcript and the miRNA-149-3p were proven in the luciferase assay and the validation was reinforced by binding site mutation experiment, using a mutated sequence of AAMP 3’-UTR in the luciferase assay (Fig 8C and 8D).

In addition, previous studies have demonstrated the ability of AAMP to interact with thromboxane A2 receptor (alpha and beta) molecules [51], which reinforce the functional activity of such receptors in vascular pathologies [52]. Furthermore, these observations help to clarify the mechanistic effects of the angiotensin-(1–7) in cardiac diseases. The heptapeptide acts in a cell, modulating the activation of thromboxane receptors, which help to control vasodilation in vascular smooth muscle [53]. Therefore, our findings on the AAMP protein level in A549 cancer cells demonstrated a decreased level of the investigated protein in Ang-(1–7) treated cells, due to the physical interaction with the miR 149-3p. If the same mechanism happens in cardiovascular tissues, it will support a decreased activation rate of thromboxane receptors, which could alleviate vasoconstriction. In cancer cells, the putative interaction of AAMP with the thromboxane receptor is connected with the poor cancer prognosis and should be investigated. This observation is based on the fact that thromboxane receptor proteins are increased in cancer cells and are connected with poor patient prognosis, because this promotes cellular proliferation and migration [51,54]. The influence of miRNA-149-3p mediated by the effect of ang-(1–7) decreases AAMP level and may be consider positive in the control of cancer cell migration. However, the considerable number of different cells and their particular metabolism should be carefully investigated before the heptapeptide is used in clinical trials. Dependent on the cell type, the strict control of gene expression and its fine-tuning takes different routes, in order to maintain cellular homeostasis. In spite of this observation, our assays found the miRNA-149-3p to be a promising element, which could support future investigation into the development of new therapeutic strategies to control cancer cell metastasis.

## Supporting Information

S1 Fig Optimal transfections conditions. Representative transfection assay using mirVana™ miRNA mimics performed with Lipofectamine® 2000 Transfection Reagents. The analyses were taken 48 h after the assays. A) Fluorescence microscopy of cellular nuclei and Alexa Fluor Red Oligo transfections. B) Gene expression analysis of *PTK9* gene after mirVana™ miRNA mimics (miR-1 Positive Control). C) Cellular viability after mirVana™ miRNA mimics (miR-1 Positive Control).
(TIF)

S2 Fig AAMP esiRNA modulates A549 cell motility and migration. A) A549 cells were grown in 6-well plates and submitted or not to RNAi using AAMP esiRNA. Under confluency, cellular migration was verified by wound healing assay and the recovery area of the injury was measured and plotted in graphs. B) Transwell chamber invasion assays of A549 cells, either submitted or not to AAMP esiRNA. Representative field of invaded and stained cells is shown (left) and the measurements were plotted in graphs. In the assays represented in A and B, ANOVA analysis found significant differences between the control and cell samples; the significance level was set at p <0.05 (***).
(TIF)
