# Correction: MicroRNA Profiling of the Effect of the Heptapeptide Angiotensin-(1-7) in A549 Lung Tumor Cells Reveals a Role for miRNA149-3p in Cellular Migration Processes

**DOI:** 10.1371/journal.pone.0190204

**Published:** 2017-12-19

**Authors:** Brenda de Oliveira da Silva, Kelvin Furtado Lima, Letícia Rocha Gonçalves, Marina Bonfogo da Silveira, Karen C. M. Moraes

In the Funding and Acknowledgement sections, the grant number from the funder Fundação de Amparo à Pesquisa do Estado de São Paulo is listed incorrectly. The correct grant number is: FAPESP 14/21645-2.
