# Supplementary figures and images for: MicroRNA Profiling of the Effect of the Heptapeptide Angiotensin-(1-7) in A549 Lung Tumor Cells Reveals a Role for miRNA149-3p in Cellular Migration Processes
Source: PLoS One. 2016 Sep 6;11(9):e0162094. doi: 10.1371/journal.pone.0162094 (PMC5012581; doi:10.1371/journal.pone.0162094)

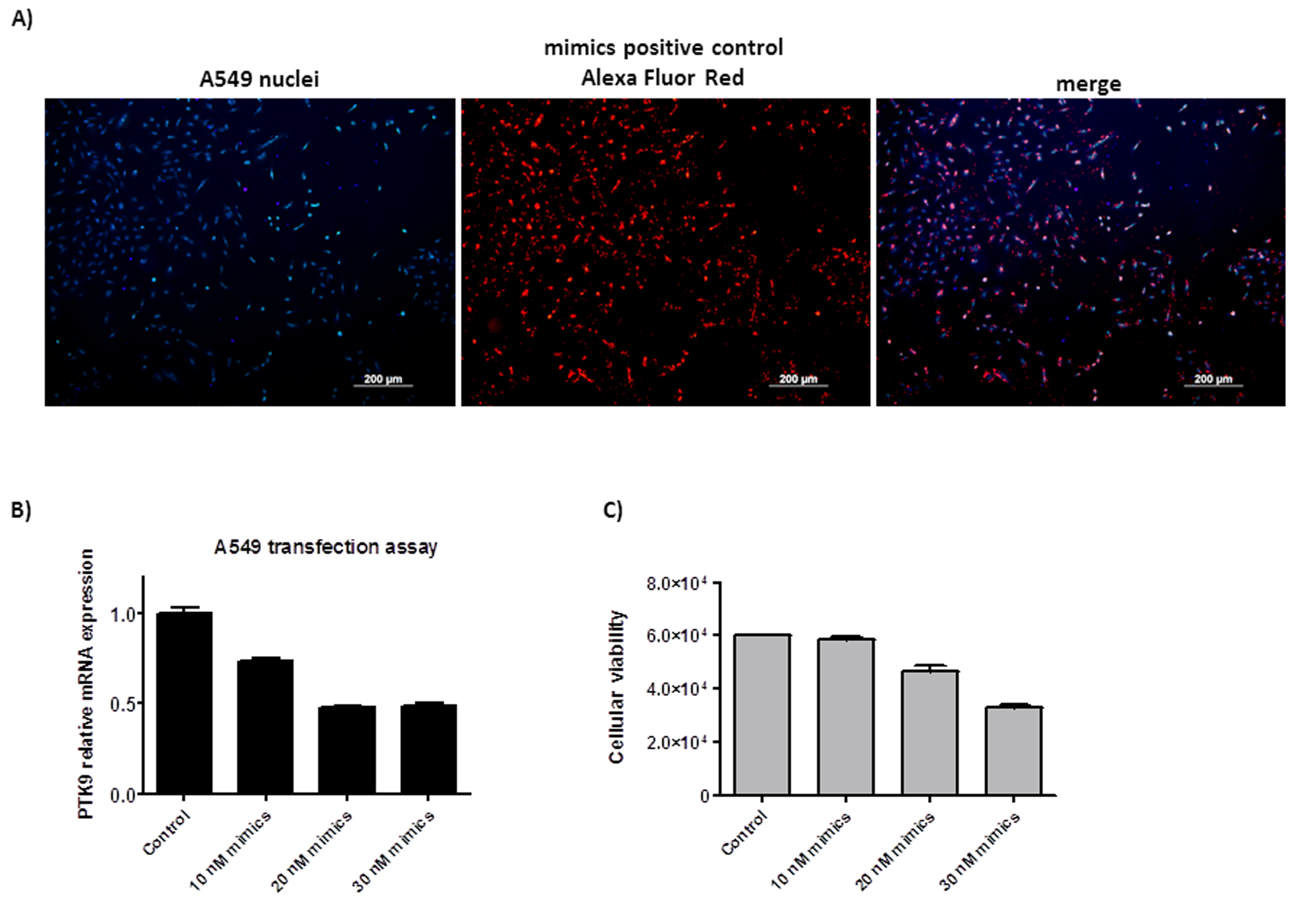

Supplement: S1 Fig — Representative transfection assay using mirVana™ miRNA mimics performed with Lipofectamine® 2000 Transfection Reagents. The analyses were taken 48 h after the assays. A) Fluorescence microscopy of cellular nuclei and Alexa Fluor Red Oligo transfections. B) Gene expression analysis of PTK9 gene after mirVana™ miRNA mimics (miR-1 Positive Control). C) Cellular viability after mirVana™ miRNA mimics (miR-1 Positive Control). (TIF) [file pone.0162094.s001.tif]

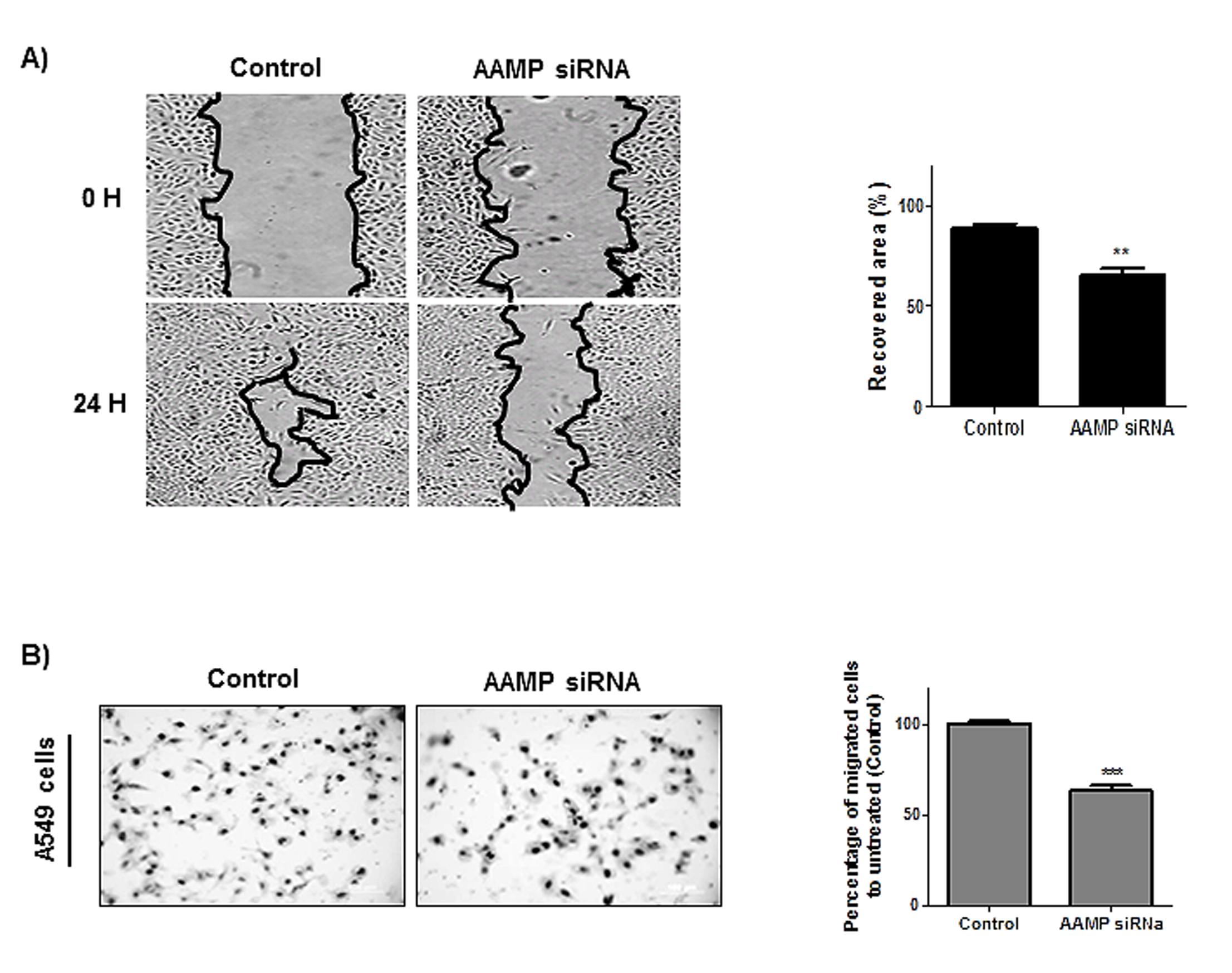

Supplement: S2 Fig — A) A549 cells were grown in 6-well plates and submitted or not to RNAi using AAMP esiRNA. Under confluency, cellular migration was verified by wound healing assay and the recovery area of the injury was measured and plotted in graphs. B) Transwell chamber invasion assays of A549 cells, either submitted or not to AAMP esiRNA. Representative field of invaded and stained cells is shown (left) and the measurements were plotted in graphs. In the assays represented in A and B, ANOVA analysis found significant differences between the control and cell samples; the significance level was set at p <0.05 (***). (TIF) [file pone.0162094.s002.tif]
